# Random Forest and Feature Importance Measures for Discriminating the Most Influential Environmental Factors in Predicting Cardiovascular and Respiratory Diseases

**DOI:** 10.3390/ijerph21070867

**Published:** 2024-07-02

**Authors:** Francesco Cappelli, Gianfranco Castronuovo, Salvatore Grimaldi, Vito Telesca

**Affiliations:** 1 DIBAF Department, University of Tuscia, 01100 Viterbo, Italy; salvatore.grimaldi@unitus.it; 2 School of Engineering, University of Basilicata, Viale dell’Ateneo Lucano 10, 85100 Potenza, Italy; vito.telesca@unibas.it

**Keywords:** Feature Importance Measures, Machine Learning, Interpretability, Public Health, Cardiovascular Diseases, Respiratory Diseases

## Abstract

Background: Several studies suggest that environmental and climatic factors are linked to the risk of mortality due to cardiovascular and respiratory diseases; however, it is still unclear which are the most influential ones. This study sheds light on the potentiality of a data-driven statistical approach by providing a case study analysis. Methods: Daily admissions to the emergency room for cardiovascular and respiratory diseases are jointly analyzed with daily environmental and climatic parameter values (temperature, atmospheric pressure, relative humidity, carbon monoxide, ozone, particulate matter, and nitrogen dioxide). The Random Forest (RF) model and feature importance measure (FMI) techniques (permutation feature importance (PFI), Shapley Additive exPlanations (SHAP) feature importance, and the derivative-based importance measure ($\kappa^{ALE}$)) are applied for discriminating the role of each environmental and climatic parameter. Data are pre-processed to remove trend and seasonal behavior using the Seasonal Trend Decomposition (STL) method and preliminary analyzed to avoid redundancy of information. Results: The RF performance is encouraging, being able to predict cardiovascular and respiratory disease admissions with a mean absolute relative error of 0.04 and 0.05 cases per day, respectively. Feature importance measures discriminate parameter behaviors providing importance rankings. Indeed, only three parameters (temperature, atmospheric pressure, and carbon monoxide) were responsible for most of the total prediction accuracy. Conclusions: Data-driven and statistical tools, like the feature importance measure, are promising for discriminating the role of environmental and climatic factors in predicting the risk related to cardiovascular and respiratory diseases. Our results reveal the potential of employing these tools in public health policy applications for the development of early warning systems that address health risks associated with climate change, and improving disease prevention strategies.

## 1. Introduction

Cardiovascular diseases (CVDs) are the leading cause of global mortality, surpassing all other health conditions [1]. Among these, ischemic heart disease and cerebrovascular disease are predominant. However, respiratory diseases (RDs), including lower respiratory infections and chronic obstructive pulmonary disease, are also major causes of death [2]. According to the Intergovernmental Panel on Climate Change (IPCC), climate change is likely to impact human health both directly, through temperature fluctuations, and indirectly, through changes in disease vectors [3] (p. 2). 

A comprehensive review of the existing scientific literature has revealed that rising temperatures will likely lead to an increase in morbidity and mortality related to weather conditions, with a significant portion of deaths linked to cardiovascular events [4,5,6]. Several studies conducted across different parts of the world have confirmed that extreme temperatures increase the risk of mortality due to CVDs and RDs [7,8,9,10,11]. It has been observed that heatwaves can cause mortality rates from cardiovascular diseases to range from 13% to 90%, cerebrovascular diseases from 6% to 52%, and respiratory diseases up to 14% [12]. In the United States, approximately 5600 heat-related deaths occurred annually from 1997 to 2006 across 297 counties [13]. Episodes like the July 2006 heatwave in California confirm the substantial incidence of emergency room visits for cardiovascular and respiratory diseases [14], recording about 140 deaths on July 15 and August 1 of the same year. Studies in nine U.S. cities identified a 1.8% increase in mortality associated with increases in apparent temperature [15]. Similarly, in North America, a 4.7 °C increase in average daily temperature was correlated with a 2.6% increase in cardiovascular mortality [16]. Furthermore, in regions where the temperature in the hottest months exceeds 30 °C, each degree increase is associated with a 3% increase in mortality [17]. In Europe too, an analysis across 15 European cities reported a strong correlation between heatwaves and mortality due to respiratory diseases [16]. Similar associations between temperature and mortality have also been observed in China, where an increase in risk occurs at both low and high temperatures [18]. Overall, analyses of daily mortality rates have highlighted that both low and high temperatures are associated with an increase in mortality from CVDs [19]. In fact, regarding CVDs, a strong positive correlation was demonstrated between maximum temperature and mortality (r = 0.83, *p* < 0.01), in addition to a significant negative correlation between minimum temperature and mortality [19]. Numerous studies have highlighted that the winter period is correlated with a marked increase in cardiovascular diseases and deaths related to them, especially in regions of the Northern Hemisphere characterized by particularly cold temperatures [10,20]. Specifically, daily rates of cardiovascular events increase with decreases in average air temperature, with a 10 °C decrease associated with a 19% increase in daily rates of cardiovascular events for individuals over 65 years [21].

These studies provide robust evidence of the health impacts of extreme temperatures, using large datasets and rigorous statistical analyses to highlight the significant increase in mortality rates during heatwaves and cold spells. However, many of these studies rely on aggregated data, which can obscure individual-level variations and the influence of other confounding factors. Additionally, they primarily focus on maximum temperatures, potentially overlooking the significant effects of minimum temperatures on health outcomes.

It must be emphasized that climate change not only affects temperature, but also has adverse effects on other environmental conditions, particularly air pollution [22]. Recent works have further explored the complex relationships between various meteorological factors (i.e., higher solar radiation, atmospheric pressure, humidity, and wind) and cardiovascular and respiratory diseases [23,24,25,26,27,28,29,30]. 

These studies underscore the limitations of frequently using linear models, which may not fully capture the complex, non-linear relationships between atmospheric pressure and health outcomes. Moreover, there is often a lack of consideration for the interaction between atmospheric pressure and other environmental factors, which could influence the results. Nevertheless, challenges related to the spatial and temporal variability of air pollution data can affect the accuracy and generalizability of findings. Additionally, the potential confounding effects of other environmental and socio-economic factors are not always fully accounted for, which can limit the robustness of conclusions.

The investigation into the relationships between meteorological factors and cardiovascular and respiratory diseases is vital, especially given the seemingly discordant views presented by existing research. Delving deeper into the influence of environmental parameters on disease patterns is crucial for illuminating specific aspects of these interactions and for enhancing our understanding of the nuanced relationships between climate, health, and disease.

Currently, an increasing amount of clinical, biometric, and biomarker data are available, and the use of artificial intelligence (AI)—and in particular machine learning (ML)—in healthcare has introduced new research perspectives and applications. These technologies’ ability to analyze vast amounts of data manifests in various areas, from more precise disease surveillance to advanced image interpretation and optimized healthcare operations management. A recent literature analysis confirmed the effectiveness of ML techniques in analyzing clinical data, offering valuable insights for early diagnosis and disease management [31,32,33]. These results suggest that ML models in specific clinical contexts outperform traditional methods, offering more accurate predictive models. Several papers [31,32,33,34,35,36,37] have delved deeply into ML applications for medical diagnoses and predictions, from cardiovascular diseases to patients’ clinical deterioration and respiratory diseases. These studies employed a variety of algorithms, including neural networks (i.e., multilayer perceptron, recurrent neural network, convolutional neural network (CNN), long short-term memory (LSTM), etc.), support vector machine, boosting methods like RF, and combinations of techniques such as CNN and LSTM, achieving high predictive performance (AUC of 0.809) [38].

In ML, understanding the importance of features is critical to clearly interpreting how the model works internally. A variety of ML techniques have been introduced in the literature to quantify feature importance both globally and locally. Specifically, the former helps to assess the global impact of features on ML model prediction, while the latter enables quantifying the specific contribution of features in each individual prediction. Feature importance measures are interpretability techniques, and belong to explainable artificial intelligence methods that help the user to understand and trust the results provided by ML models.

In healthcare and water science, a variety of these techniques have been applied, proving their potential [39,40,41,42,43]. Specifically, Ref. [42] offered a comparison of a multitude of global importance measures by considering several case studies that can mimic real-world hydrological scenarios. This analysis shows that the most robust feature importance measures are permutation feature importance (PFI) [44], Shapley Additive exPlanations (SHAP) feature importance [45,46], and the derivative-based importance measure ($\kappa^{ALE}$) [41].

A study conducted at the Policlinico Giovanni XXIII in Bari (Italy) [47] highlighted AI’s ability to model correlations between climatic conditions and the incidence of CVDs. Using feature importance techniques derived from the RF algorithm, meteorological variables such as average, maximum, and apparent temperature, along with relative humidity, were identified as key indicators of hospitalizations related to CVDs. 

Starting from the analysis conducted in [47], our goal is to extend the previous research by applying a set of feature importance measures that have proven to be robust and effective in identifying key factors in hydrological applications [42]. We compare the results to gain a comprehensive view of which factors most affect the risk of mortality due to cardiovascular and respiratory diseases. 

Our study builds on this existing body of research by addressing some of the limitations identified in the literature. By employing the Random Forest (RF) model, we handle complex, non-linear relationships and interactions between multiple environmental factors. The integration of Seasonal Trend Decomposition using LOESS (STL) for data pre-processing effectively isolates trend components from seasonal and irregular noise, enhancing the robustness and accuracy of our predictions. Furthermore, our use of feature importance measures (PFI, SHAP, *κ^ALE^*) allows for a flexible and comprehensive analysis that is adaptable to various machine learning models and datasets. This approach not only enhances predictive accuracy, but also improves model interpretability, providing actionable insights for policymakers and healthcare providers.

### Contributions of the Study

This study makes several significant contributions to the field of environmental health and predictive modeling. By employing permutation feature importance (PFI), Shapley Additive exPlanations (SHAP), and the derivative-based importance measure ($\kappa^{ALE}$), this study identifies the most influential climatic factors in predicting cardiovascular (CVD) and respiratory (RD) disease admissions. The findings highlight the critical roles of atmospheric pressure, minimum temperature, and carbon monoxide levels in health outcomes. The integration of Seasonal Trend Decomposition using LOESS (STL) for data pre-processing represents a novel approach in this context. STL effectively isolates trend components from seasonal and irregular noise, thereby enhancing the robustness and accuracy of predictive models. The use of Random Forest (RF) models to handle complex, non-linear relationships and interactions between multiple environmental factors demonstrates the effectiveness of advanced machine learning techniques in public health research. The study employs feature importance measures, providing a flexible and comprehensive analysis that is adaptable to various machine learning models and datasets. This approach not only improves predictive accuracy, but also enhances model interpretability, offering actionable insights for policymakers and healthcare providers. The identification of key environmental predictors allows for the development of more accurate early warning systems. These systems can anticipate spikes in disease admissions, enabling timely public health interventions and informing targeted strategies to mitigate the health impacts of climate change. These contributions collectively advance our understanding of the relationship between environmental factors and health outcomes, offering new perspectives and methodologies for future research in the field.

## 2. Methods

### 2.1. Design and Setting of the Study

In this study, daily admissions to the emergency room for cardiovascular and respiratory diseases are jointly analyzed with daily environmental and climatic parameter values (temperature, atmospheric pressure, relative humidity, carbon monoxide, ozone, particulate matter, and nitrogen dioxide). The main aim is to investigate the role of each factor in admission prediction and to identify the most influential environmental factors on disease development.

**Figure 1:**
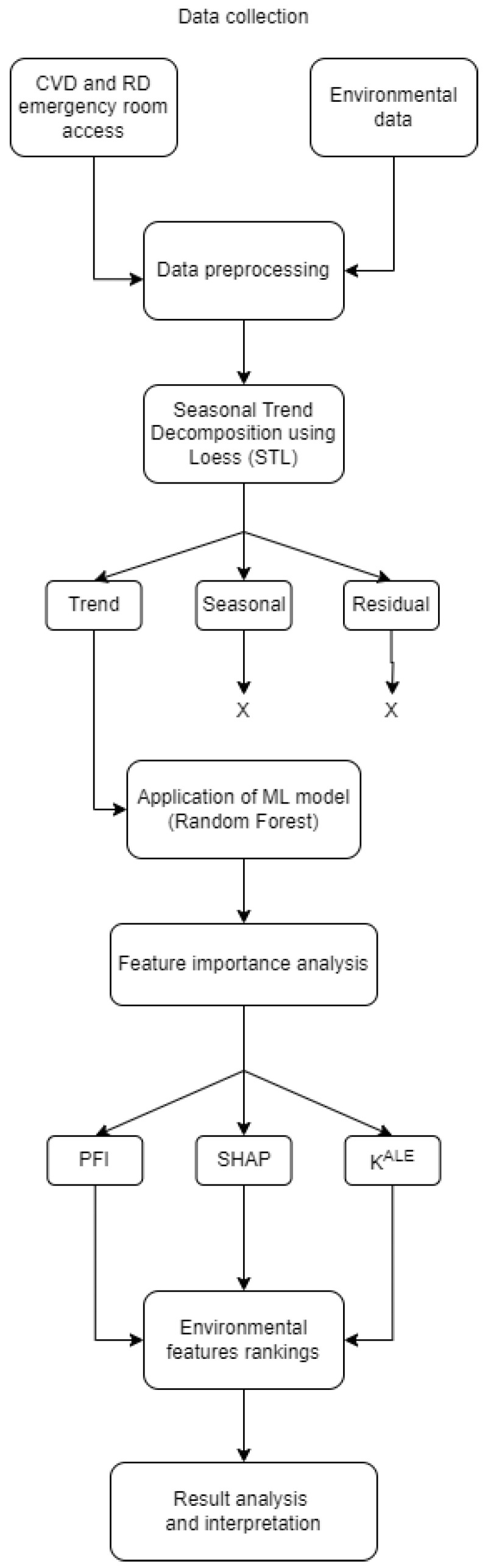
Flowchart of research methodology. The process begins with collecting daily data on admissions for cardiovascular and respiratory diseases, along with daily environmental and climatic parameters. The raw data undergo a pre-processing step and the STL (Seasonal Trend Decomposition using Loess) method is employed to remove residual and seasonal behaviors. Subsequently, a Random Forest model is applied to predict disease admissions based on the pre-processed environmental data. Feature importance measures, including permutation feature importance (PFI), SHapley Additive exPlanations (SHAP), and the derivative-based importance measure ($\kappa^{ALE}$), are then computed to analyze and identify the most influential environmental parameters.

To provide a clear overview of the research process, Figure 1 illustrates the main steps of the methodology used in this study. The flowchart outlines the sequential approach from data collection to the interpretation of results, highlighting the key phases of the research.

In our application, we partition the data into 80% for training and 20% for testing. The data are pre-processed to remove trend and seasonal behavior using the STL method and preliminary analyzed to avoid redundancy of information. In the proposed case study, a preliminary analysis revealed that the data were not affected by missing values or outliers. However, we strongly recommend addressing such issues to ensure they do not compromise the quality or significance of the analysis.

### 2.2. Feature Importance Measures 

In the present study, we apply three ML feature importance measures: permutation feature importance (PFI), Shap feature importance (Shap), and the derivative-based importance measure ($\kappa^{ALE}$). These are model-agnostic tools, as they can be applied to any supervised ML algorithm, such as linear models, RFs, gradient boosting, or neural networks [48]. These techniques exhibit the advantages of not relying on specific assumptions regarding (a) the nature of the relationship (linear or non-linear) between the features and the output response or (b) the distribution of the data. 

As a general notation, we consider the ML prediction function $\hat{f}:R^{d}\to R,$ where $f\left(x\right)$ is a model prediction and x is a d-dimensional feature vector. Let $X_{j}$ and $x_{j}\in R^{n}$ be the *j*-th feature as a random variable and an observed feature, respectively. Let $X_{j}$ be the support of $X_{j}$ and let $X_{-j}=X\backslash X_{j}$ be the complementary support of $X_{-j}=\left\{X_{k}:k=1,\ldots ,d;k\neq j\right\}.$ We denote the value of the *j*-th feature from the *i*-th observation by $x_{j}^{\left(i\right)}$ and the associated target value by $y^{\left(i\right)}.$ We refer to the training data of the ML model with ${\left\{\left(x^{\left(i\right)},\;y^{\left(i\right)}\right)\right\}}_{i=1}^{n}$.

Permutation feature importance (PFI) was introduced by [44]. It is defined as

(1) $$PFI_{j}=E\left[L\left(Y,\hat{f}\left(X_{j}^{\pi \;},\;X_{-j}\right)\right)\right]-E\left[L\left(Y,\hat{f}\left(X_{j}^{\;},\;X_{-j}\right)\right)\right]\;\;\;$$

This measure quantifies the importance of a feature based on the discrepancy between the expected loss when a feature $X_{j}$ is permuted and the original loss. PFI is estimated with the following formula:

(2) $$\hat{PFI_{J}}=\frac{1}{n}\overset{n}{\underset{i=1}{\sum }}L\left(y^{\left(i\right)},\;\hat{f}\left(x_{j}^{\pi \left(i\right)},\;x_{-j}^{\left(i\right)}\right)\right)-\frac{1}{n}\overset{n}{\underset{i=1}{\sum }}L\left(y^{\left(i\right)},\;\hat{f}\left(x^{\left(i\right)}\right)\right).\;\;\;$$

SHAP feature importance is built on the notion of Shapley values introduced in cooperative game theory by [45]. Consider a game with a group of players represented by the set $D=\left\{1,\ldots ,d\right\}$, where d is the total number of players. They can form coalitions K⊆D. Now, we define a value function $v:\;2^{d}\to R_{+},$ where $2^{d}$ represents the set of all possible coalitions of the players. The function v assigns a non-negative real number to each subset of players, which corresponds to their recompense. The reward of the j-th player is

(3) $$\phi_{j}\left(v\right)=\underset{K\subseteq P\backslash \left\{j\right\}}{\sum }\frac{\left|K\right|!\left(\left|D\right|-\left|K\right|-1\right)!}{\left|D\right|!}\;\left[v\left(K\cup \left\{j\right\}\right)-v\left(K\right)\right],\;$$

where the difference between the value functions quantifies the marginal contribution of the j-th player in coalition K. Shapley’s value allows rewards to be allocated fairly among players, assuming they cooperate.

Ref. [46] introduced a value function $v_{\hat{f}}\left(K\right)$ based on an ML model $\hat{f}$. It is defined as the conditional expectation of the dependent feature (target) in a specific observation when the features in coalition K are known. The value function $v_{\hat{f}}\left(K\right)$ is defined as follows:

(4) $$v_{\hat{f}}\left(K\right)=E\left[X_{K}=x_{K}\right]=E_{X_{-K}|X_{K}}\left[\hat{f}\left(x_{K}\right),X_{-K}\right].\;$$

SHAP feature importance is defined by averaging the absolute Shapley values per feature across the data:

(5) $$SHAP_{j}=\frac{1}{n}\overset{n}{\underset{i=1}{\sum }}\left|\phi_{j}^{\left(i\right)}\right|.\;$$

The derivative-based importance measure ($\kappa^{ALE}$), recently introduced by [41], is computed using the ALE plot design [49]. ALE plots are interpretable ML tools that belong to the class of Feature Effect indicators. These plots provide insights on the marginal effect of a feature on the target. Specifically, ALE plots visualize the direction and magnitude of the impact of a specific feature on ML model prediction. To estimate an ALE function, one has to split the support $X_{j}$ of $X_{j}$ into K at mutually exclusive and exhaustive intervals, i.e., $X_{j}^{K}=\left(z_{j}^{k-1},z_{j}^{k}\right]$, with k=1, …, K. The $ALE_{j}\left(x_{j}\right)$ is estimated by the following [49]:

(6) $${\hat{ALE}}_{j}\left(x_{j}\right)=\overset{K}{\underset{k=1}{\sum }}\frac{1}{n_{j}^{k}}\underset{i:\;x_{i}\in X_{j}^{k}}{\sum }\;\left[\hat{f}\left(z_{j}^{k},\;x_{-j}^{\left(i\right)}\right)-\hat{f}\left(z_{j}^{k-1},\;x_{-j}^{\left(i\right)}\right)\right],\;$$

for each $x_{j}\in$ $=\left(z_{j}^{0},z_{j}^{K}\right]$, where $z_{j}^{0}=\left\{x_{j}^{\left(1\right)},\ldots ,x_{j}^{\left(n\right)}\right\}$ and $z_{j}^{K}=\left\{x_{j}^{\left(1\right)},\ldots ,x_{j}^{\left(n\right)}\right\}\;.$ Note that ALE functions are built by exploiting the sum of the local effects for all observations falling in a neighborhood of x. 

The derivative-based importance measure $\kappa^{ALE}$ of $X_{j}$ is defined as

(7) $$\kappa_{j}^{ALE}=\frac{1}{K}\overset{K-1}{\underset{k=0}{\sum }}E{\left[\frac{\hat{f}\left(X_{j}^{k},\;X_{-j}^{\left(i\right)}\right)-\hat{f}\left(X_{j}^{k-1},\;X_{-j}^{\left(i\right)}\right)}{X_{j}^{k}-X_{j}^{k-1}}\right]}^{2}\frac{\sigma_{X_{j}}^{2}}{\sigma_{Y}^{2}},\;\;\;$$

where $\sigma_{X_{j}}^{2}$ and $\sigma_{Y}^{2}$ are variances of the j-th feature and the target, respectively. It can be estimated by

(8) $${\hat{\kappa }}_{j}^{ALE}=\frac{1}{K}\overset{K-1}{\underset{k=0}{\sum }}\underset{i:\;x^{\left(i\right)}\in X_{j}^{k}}{\sum }\;{\left[\frac{\hat{f}\left(z_{j}^{k},\;x_{-j}^{\left(i\right)}\right)-\hat{f}\left(z_{j}^{k-1},\;x_{-j}^{\left(i\right)}\right)}{z_{i}^{k}-z_{i}^{k-1}}\right]}^{2}\frac{{\hat{\sigma }}_{x_{j}}^{2}}{{\hat{\sigma }}_{y}^{2}}.$$

The proposed measure is a normalized expectation of Newton ratios computed at randomized locations in the feature space in the spirit of derivative-based sensitivity measures presented in [50].

The importance analysis is performed using the R-package *vip* [51] to compute PFI and Shap importance measures, and an R function is built by one of the authors to compute the $\kappa^{ALE}$ importance measure.

### 2.3. Machine Learning Model: Random Forest 

The feature importance measures introduced in the previous section are computed by leveraging the predictive ability of an ML model. Among the various supervised ML models available in the literature, we selected the RF model for our study to replicate the analysis conducted in [47].

RF is a powerful ensemble learning method employed for classification and regression tasks. It builds multiple decision trees during training, with each tree constructed on a data sample extracted from a training set. The output of RF is the class selected from most trees for classification tasks or the average prediction of individual trees for regression tasks. This model includes two main hyperparameters: the number of trees (*n.trees*) and the number of features sampled for splitting at each node (*mtry*). For a more in-depth exploration of the theoretical foundations of the RF algorithm, refer to [52,53]. To achieve a high accuracy with the ML model, we employ a combination of grid-search and cross-validation methods to help us in finding the optimal hyperparameters [54,55]. The grid-search method is a common approach used to tune hyperparameters in ML models. It requires the user to define a predefined grid of hyperparameter combinations to be explored. For each combination, a separate ML model is built and evaluated. Cross-validation is a technique to assess the performance and the ability of an ML model. An available dataset is divided into *k* training and validation subsets, where k is typically chosen as a parameter. In our analysis, *k* is set to 5. The ML model is trained and evaluated multiple times, with each subset serving as the validation set once. This process helps to obtain a more robust estimate of the model’s performance. 

The grid-search method combined with cross-validation allows us to systematically evaluate different model configurations by training and evaluating multiple models with different hyperparameter combinations. The validation error, computed by averaging the performance measures obtained from all *k* subsets, is used as the criterion for selecting the optimal model configuration. 

The RF algorithm is used in several fields (such as water science, finance, and healthcare, just to mention a few) due to its flexibility. It has proven to be (a) effective in handling non-linear and complex relationships among features, (b) robust against overfitting and outliers, and (c) adept at managing heterogeneous data and collinearity [44]. 

However, the RF model is considered a “black box” model because of the complexity of its structure, especially when the number of decision trees increases. This makes the inner workings of the model non-transparent [48]. To correctly interpret the results of ML models, diagnostic tools (such as feature importance measures, marginal effect indicators, etc.) are required. A limitation to the use of such a model is dictated by computational complexity. Building such a model with a large number of trees and high-dimensional data can be computationally expensive and time-consuming. In general, RF can be considered a powerful and widely used ML model in the literature because of its characteristics. 

The analysis in our study is conducted using the following R packages: *randomForest* [53] to implement the ML model, and *caret* [56] to perform hyperparameter optimization.

### 2.4. Seasonal Trend Decomposition

To enhance the prediction accuracy of the ML model and improve the reliability of the importance analysis, trends, seasonal variations, and irregularities in the data should be removed. With this aim, we include in our study Seasonal Trend Decomposition using LOESS (STL), a technique well known in the literature [57,58,59] that offers significant advantages in the analysis and interpretation of temporal data. The STL technique dissects complex time series data into distinct, interpretable components [60]. It decomposes the time series into trend, seasonal and residual elements, allowing a clearer and complete picture of the data. This approach is particularly useful when dealing with data with inherent seasonal variations or when the focus is on long-term trends [61]. By using STL, we can uncover underlying trends and patterns that might be obscured in the original analysis, thus providing a more in-depth view of the data [62]. In general, the use of the STL method is crucial for conducting rigorous and accurate analysis, as it helps to extract meaningful information from temporal data, improve forecasts, and facilitate a more robust significance analysis. STL operates on the principle of additive decomposition, i.e., breaking down a time series into a sum of its constituent components: the trend component $T_{v}$, representing the long-term direction of the series; the seasonal component $S_{v}$, capturing cyclical variations at regular intervals; and the residual component $R_{v}$, encompassing the unexplained variance.

(9) $$Y_{v}=T_{v}+S_{v}+R_{v}.\;$$

The use of LOESS in STL, a non-parametric regression method, further refines the analysis by estimating the trend and seasonal components based on local data behavior [63]. LOESS (Locally Estimated Scatterplot Smoothing) is particularly advantageous because it does not rely on assumptions of linearity or stationarity, which are often limiting when dealing with complex environmental data that exhibit non-linear and non-stationary behaviors. This flexibility enables LOESS to provide a more accurate representation of underlying patterns, enhancing the robustness of model comparisons.

In contrast, Fourier analysis decomposes a time series into sinusoidal components and is better suited for identifying periodic patterns. However, its reliance on linearity and stationarity assumptions makes it less effective for our purposes. Moving averages, while useful for short-term smoothing, can obscure significant variations and trends due to their simplicity. LOESS improves upon this by locally fitting polynomial regressions within overlapping windows, capturing more intricate patterns without oversimplifying the data. The practical application of LOESS in our study involved reprocessing the original environmental data to emphasize the trend component, thereby removing the seasonal and irregular noise. This step was instrumental in clarifying the data’s structure, making the underlying trends more apparent, and facilitating a more accurate interpretation of the relationships between environmental factors and health outcomes such as CVD and RD. The subsequent recalibration of the correlation matrix post-STL application yielded enhanced Pearson coefficient values, highlighting the efficacy of this method in uncovering more pronounced correlations between environmental variables and target health outcomes. In summary, LOESS offers significant advantages over Fourier analysis and moving averages by providing a flexible, non-parametric approach to trend estimation. This leads to improved model comparison and a deeper understanding of the data, ultimately enhancing the reliability and applicability of our findings in environmental health research.

This feature of STL is crucial for adapting to the complexity of environmental data, which often exhibit non-linear trends and seasonal fluctuations [64]. The application of STL in this study involved reprocessing the original environmental data to emphasize the trend component, thereby removing the seasonal and irregular noise. This step was instrumental in clarifying the data’s structure, making the underlying trends more apparent, and facilitating a more accurate interpretation of the relationships between environmental factors and health outcomes such as CVD and RD. The subsequent recalibration of the correlation matrix, post-STL application, yielded enhanced Pearson coefficient values, highlighting the efficacy of this method in uncovering more pronounced correlations between environmental variables and target health outcomes. This enhanced understanding is pivotal for developing predictive models and formulating hypotheses in public health research, demonstrating the transformative impact of advanced statistical techniques in elucidating complex relationships within environmental health data. The use of LOESS in STL, a non-parametric regression method, further refines the analysis by estimating the trend and seasonal components based on local data behavior. This feature of STL is crucial for adapting to the complexity of environmental data, which often exhibit non-linear trends and seasonal fluctuations. The application of STL in this study involved reprocessing the original environmental data to emphasize the trend component, thereby removing the seasonal and irregular noise. This phase plays a crucial role in elucidating the structure of the data, bringing out underlying trends, and enabling a more accurate interpretation of the relationships between environmental factors and health outcomes such as CVD and DR.

### 2.5. Performance Indices

To comprehensively evaluate the performance of the ML model, we employ three distinct indices: mean absolute error (MAE), mean absolute relative error (MARE), and the coefficient of determination ($R^{2}$).

By evaluating these three performance metrics, we obtain a complete overview of the ML model’s performance, considering both absolute and relative errors, as well as the overall goodness of fit. 

MAE is defined as

(10) $$MAE=\frac{1}{n}\;\overset{n}{\underset{i=1}{\sum }}\left|y_{i}-{\hat{y}}_{i}\right|,\;\;\;$$

where y represents the vector of observed target values and $\hat{y}$ corresponds to the vector of predicted values. This index reflects the mean of the absolute differences between observed and predicted values. 

MARE is determined as the average of the absolute differences between the observed and predicted values divided by the observed values, i.e.,

(11) $$MARE=\frac{1}{n}\overset{n}{\underset{i=1}{\sum }}\frac{\left|y_{i}-{\hat{y}}_{i}\right|}{y_{i}}.\;$$

The third performance metric, the coefficient of determination ($R^{2}$), is expressed as

(12) $$R^{2}=1-\frac{\sum_{i=1}^{n}{\left(y_{i}-{\hat{y}}_{i}\right)}^{2}}{\sum_{i=1}^{n}{\left(y_{i}-{\underline{y}}_{i}\right)}^{2}},\;\;$$

where $\underline{y}$ represents the mean value of y. The index $R^{2}$ quantifies the percentage of variation in the output variable elucidated by the ML model predictions. Note that the MAE metric spans the range from 0 to ∞, where 0 indicates a perfect fit, while MARE and $R^{2}$ metrics are expressed in relative terms, so these performance indices span from 0 to 1.

Using these performance measures, namely MAE, MARE, and $R^{2}$, provides a comprehensive and balanced evaluation of the predictive model. MAE captures the average absolute error between the model’s predictions and the actual values, allowing for a direct assessment of accuracy. MARE complements MAE by considering the relative error in relation to the actual values, providing insights into proportional errors in certain contexts. On the other hand, R^2^ indicates how well the model fits the data by measuring the proportion of total variance explained by the model.

By incorporating these measures together, a more holistic understanding of the predictive model’s performance can be achieved. MAE and MARE offer detailed insights into the model’s absolute and relative accuracy, respectively, while $R^{2}$ provides an indication of the overall fit to the data. This combination allows for a comprehensive evaluation of the model’s capabilities and limitations, enabling researchers and practitioners to make informed decisions regarding its use.

### 2.6. Case Study: Database Description and Data Pre-Processing

The data used in this study regard daily emergency room admissions at the Policlinico Hospital in Bari, covering the period from 2013 to 2021. The database of daily admissions categorized the primary problem, i.e., the pathology presented by patients upon arrival at the emergency room. Out of the 33 categories of pathologies identified in the emergency admissions, only those related to cardiovascular and respiratory diseases were selected for analysis.

Data on the meteorological and climatic conditions of Bari for the period 2013–2021 were sourced from the Arpa Puglia website and the Meteonetwork measurement network. Arpa Puglia manages two monitoring networks: one consisting of 5 automatic stations located at its provincial offices (Bari, Brindisi, Foggia, Lecce, and Taranto) and a second one, the meteorological network, supplementary to the air quality monitoring network (RRQA), currently comprising 19 stations. Data on Bari’s air quality from 2013 to 2021 were also obtained from the Arpa Puglia website, focusing on the Bari—Caldarola, Bari—CUS, Bari—Kennedy, and Bari—Carbonara stations through a mobile laboratory. The meteorological and climatic data were recorded at a half-hourly frequency for the Arpa Puglia meteorological stations and at a frequency of every five minutes and hourly for the Meteonetwork measurement network, with the hourly interval being specific to the years 2020 and 2021, while the air quality data were recorded daily. The meteorological and climatic parameters considered in the analysis include average daily minimum, maximum, and mean temperatures (*Tmin*, *Tmax*, *Tmean*), average daily dew point temperature (*Tdewp*), average daily atmospheric pressure (*P_atm*), and average daily relative humidity (*rh*), while the air quality parameters are carbon monoxide (*CO*), ozone (*o3*), particulate matter (*pm10*), and nitrogen dioxide (*NO2*).

## 3. Results

**Figure 2:**
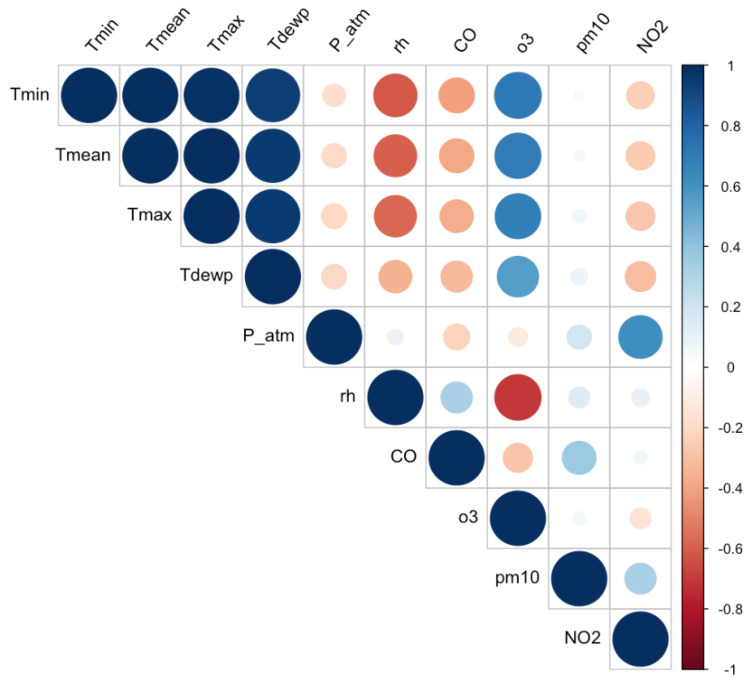
Heatmap of environmental factors.

Before conducting the feature importance analysis, we examined the data for multicollinearity, which refers to the presence of redundant features. In such cases, it is crucial to remove highly correlated features to enhance the quality and efficiency of the proposed analysis, since feature importance measures could be sensitive to it. This process facilitates the following: (a) reducing ML model complexity, thereby improving the interpretability of the findings; (b) improving the accuracy of ML predictions, thereby enabling a more robust importance analysis. To assess multicollinearity, we computed the correlation matrix using Pearson correlation coefficients (Figure 2). 

The correlation coefficients along the diagonal are all equal to 1, since each feature is perfectly correlated with itself. From the heatmap in Figure 2, we observe, as expected, that *Tmin*, *Tmean*, *Tmax*, and *Tdewp* are strongly correlated with each other. To avoid the problem of multicollinearity ([65]), we decided to focus on only *Tmin* in our analysis. Consequently, the final set of features involved in this work included three key meteorological variables: average daily minimum temperature (*Tmin*), average daily atmospheric pressure (*P_atm*), and average daily relative humidity (*rh*); and four air quality parameters: carbon monoxide (*CO*), ozone (*o3*), particulate matter (*pm10*), and nitrogen dioxide (*NO2*). 

Table 1 provides a comprehensive summary of the statistics of the seven selected environmental factors and the two disease indices, including mean, standard deviation, minimum, 25th percentile, median, 75th percentile, and maximum for each feature.

The minimum temperature (*Tmin*) shows a range from a low of −0.17 °C to a high of 32.86 °C, with an average value of 17.45 °C, indicating a broad spectrum of thermal conditions. Atmospheric pressure (*P_atm*) is observed to vary between 976.60 and 1033.67 hPa, reflecting typical fluctuations in weather patterns. Relative humidity (*rh*), an important factor in both comfort and health, is recorded with values ranging from 25.49% to 99.00%, with an average of 70.61%, illustrating diverse humidity conditions. Carbon monoxide (*CO*) levels, a critical pollutant, vary from a minimum of 0.10 ppm to a maximum of 3.00 ppm, with an average of 0.84 ppm, suggesting varied exposure levels in the studied environment. Ozone (*o3*) concentrations, a significant component of air quality, range from 13.00 to 154.00 µg/m^3^. Particulate matter (*pm10*), a key air pollutant, shows a range from 2.00 to 117.00 µg/m^3^, indicating varying degrees of air quality. Nitrogen dioxide (*NO2*) levels, another crucial air pollutant, vary from 5.00 to 157.00 µg/m^3^. The cardiovascular and respiratory disease rates, measured as cases per unit population, show a range of 0 to 37 for CVD and 0 to 25 for RD, providing insights into the health impacts associated with these environmental parameters.

Since the environmental dataset is affected by trends that influence the dependence on features, we apply the STL technique to enhance the information present in the data, as undertaken in previous research works [47,66].

**Figure 3:**
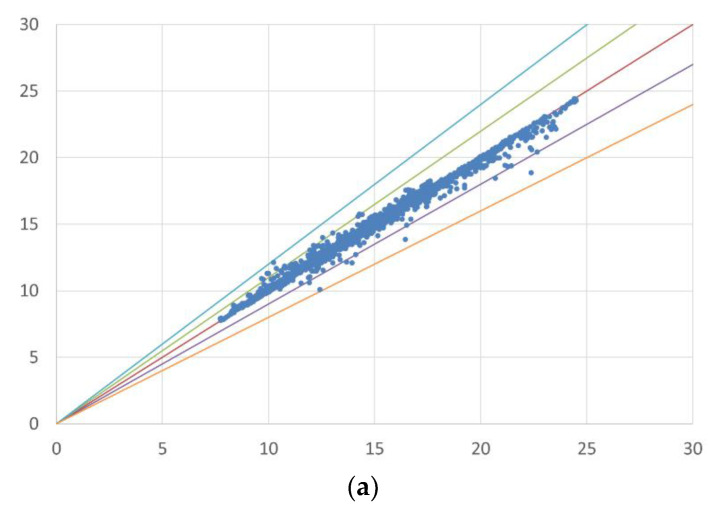

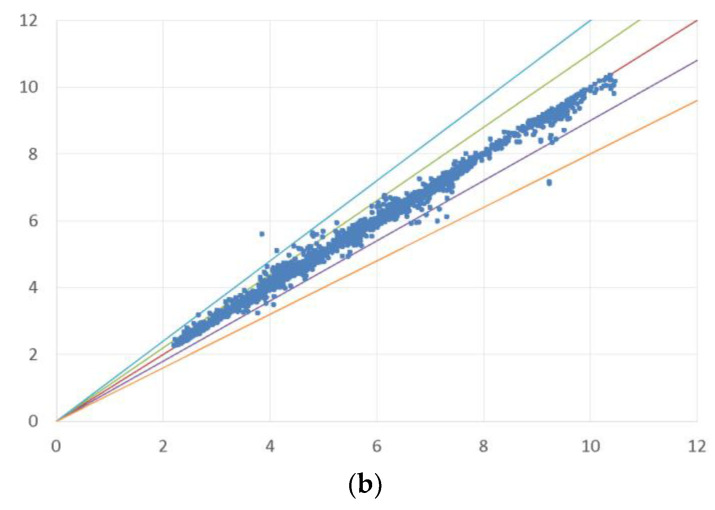
Error distribution (error bands) between actual and simulated data. The first chart (**a**) presents a scatter plot comparing simulated and actual values in the case of CVD, while the second chart (**b**) does the same for RD. The red line is the bisector of the chart, representing a perfect match between actual and simulated data. The green and purple bands indicate an error of + and −10%, respectively, whereas the blue and orange bands represent an error of + and −20%.

Predictions using the RF model on the filtered dataset provide low mean absolute errors for cardiovascular (MAE = 0.12) and respiratory diseases (MAE = 0.17), and most actual and predicted data fall within a 10% error margin, rarely exceeding 20% (Figure 3).

The three feature importance measures are applied by exploiting the predictive ability of the RF model in both target cases: CDV and RD. The estimates are normalized to facilitate the comparison between the importance measures and to ease the identification of the most significant features.

**Figure 4:**
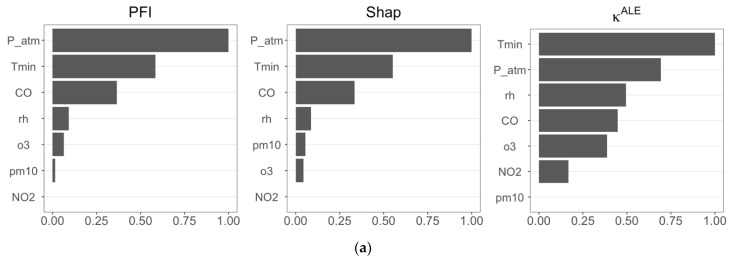

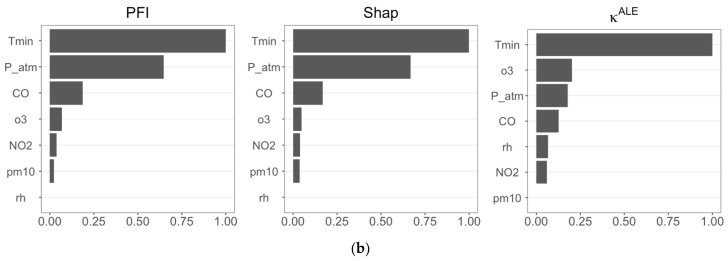
(**a**) Estimates of the three FIMs (PFI, Shap, and $\kappa^{ALE}$) calculated using RF forecasts considering CVD as the target variable (Case 1); (**b**) estimates of the three FIMs (PFI, Shap, and $\kappa^{ALE}$) calculated using RF forecasts considering RD as the target variable (Case 2).

Figure 4 reports the results of feature importance analyses for Cases 1 and 2, and, in general, we observe that the three feature importance measures provide similar results.

In both scenarios (Figure 4a,b), PFI and Shap feature importance are in perfect agreement, identifying *P_atm*, *Tmin*, and *CO* as the most influential features in predicting CVD and RD. The importance measure $\kappa^{ALE}$ differs, selecting *Tmin, P_atm*, and *rh* as the most important features in Case 1, and including *o3* in Case 2. We observe in Figure 4b that $\kappa^{ALE}$ recognizes that *Tmin* has a dominant role in RF prediction, as also confirmed by PFI and Shap feature importance. 

## 4. Discussion

FIMs are a particularly useful ML technique since they provide a ranking of importance in a given set of features, and this is crucial for understanding the contribution of individual features to overall ML predictive performance. Each FIM is associated with a corresponding importance ranking. A viable strategy is to consider the average of individual rankings [42].

In the present study, we define incremental configurations to analyze the predictive accuracy of the RF model. This approach involves constructing multiple RF model configurations (called ‘configurations’) by gradually including one variable at a time according to the resulting importance ranking obtained from a specific FIM. Specifically, ‘*conf1*’ identifies ML models that include only the first-ranked influential variable as a regressor, ‘*conf2*’ includes the two most influential variables, and so on. 

This approach helps to investigate and enables us to understand how the performance of the ML model evolves with different configurations. By evaluating the model’s predictive accuracy at each step, we can identify the features that have a more significant impact on overall performance. This allows us to determine which variables are crucial for achieving higher predictive accuracy and which ones may have a lesser influence.

In the present analysis, we avoid creating a unique average ranking because the three importance measures differ in nature and provide distinct insights. By considering each measure separately, we aim to avoid obscuring important information and ensure that the heterogeneity across the measures is captured appropriately. By examining the results independently, we can highlight the specific contributions and interpretations offered by each feature importance measure, providing a comprehensive and detailed analysis. This strategy will enhance the transparency and comprehensibility of our findings, enabling readers to gain a deeper understanding of the importance rankings resulting from each feature importance measure. As described in Section 2.5, we employ three criteria to evaluate the RF performance: MAE, MARE, and $R^{2}$. 

**Figure 5:**
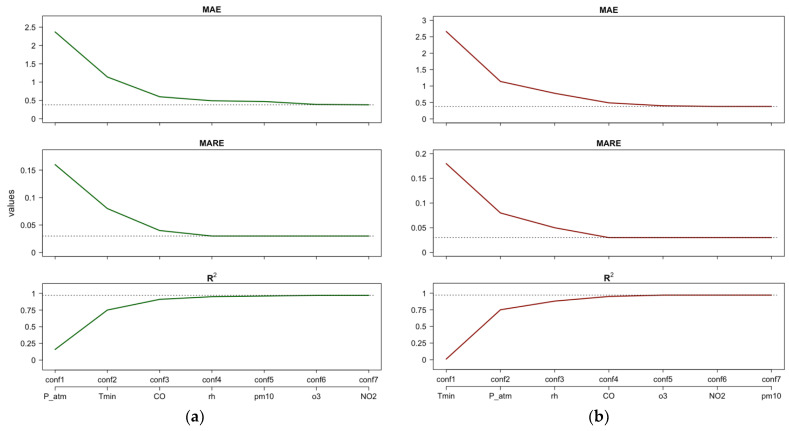
CVD—Case 1: Estimates of performance indices resulting from the incremental configurations (‘conf’) of RF constructed using PFI/Shap importance ranking (**a**) and the $\kappa^{ALE}$ importance ranking (**b**). Horizontal lines indicate the best performance achieved by the full RF model after tuning.

Figure 5 shows the performance indices of the fitted RF model assuming CVD as the target when the ML configurations are estimated using the importance ranking resulting from PFI or Shap feature importance (Figure 5a), and the importance ranking resulting from $\kappa^{ALE}$ importance measure (Figure 5b). Note that including only *P_atm* results in a better performance of the RF model (i.e., *conf1* in Figure 5a) than including only *Tmin* (i.e., *conf1* in Figure 5b).

Figure 5a,b suggest that focusing only on *P_atm*, *Tmin*, *CO*, and *rh* yields a performance close to the best performance obtained with the full RF model. For the “full” RF model, we refer to the case in which all regressors are included (*conf7*). From the first to the third configuration (Figure 5a), we observe that the performance indices MAE and MARE decrease by approximately 80%, and $R^{2}$ increases by approximately five times. Moreover, from the third to the fourth configuration (Figure 5a), we observe a residual improvement in RF performance: the MAE and MARE decrease by 18% and 25%, respectively, and $R^{2}$ increases by 4%.

**Figure 6:**
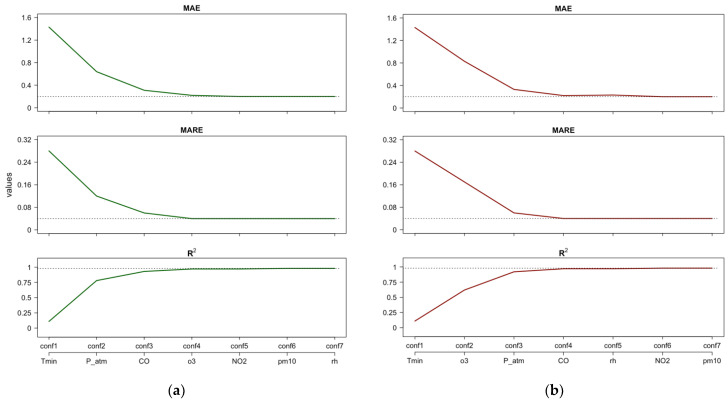
RD—Case 2: Estimates of performance indices resulting from the incremental configurations (‘conf’) of RF constructed using the PFI/Shap importance ranking (**a**) and the $\kappa^{ALE}$ importance ranking (**b**). Horizontal lines indicate the best performance achieved by the full RF model after tuning.

Figure 6, similar to Figure 5, shows the performance evolution of the RF model assuming RD as the target. In Figure 6a, the configurations refer to the PFI or Shap importance rankings, while Figure 6b similarly refers to $\kappa^{ALE}$. In this case, both scenarios in Figure 6 suggest that four features have the greatest impact in RF model predictions: *Tmin*, *P_atm, CO*, and *o3*.

**Figure 7:**
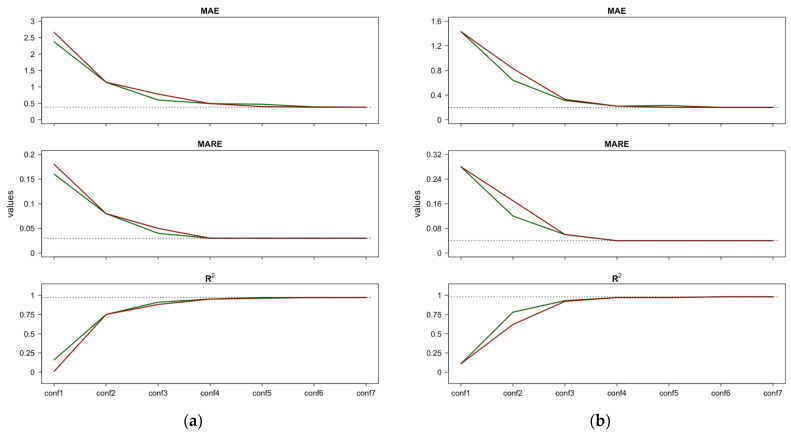
Comparison between the performances resulting from the incremental configurations (‘conf’) of RF constructed using the PFI/Shap importance ranking and those resulting using the importance ranking for CVD—Case 1 (**a**) and CVD—Case 2 (**b**). Horizontal lines indicate the best performance achieved by the full RF model after tuning.

As mentioned above, we decided to consider the importance rankings individually because each of the three importance measures captures different details; indeed, they differ in feature ordering. This discrepancy is due to the expected uncertainty of the importance measures. Using the average of individual importance rankings, as proposed in [41], could undermine any peculiarities highlighted by the importance measures. In this regard, a straightforward approach to selecting the preferred importance ranking could be comparing the slopes of the performance curves obtained with each importance ranking and choosing the one that exhibits a faster decay (for MAE and MARE) or improvement (for $R^{2}$). This strategy is shown in Figure 7, where we compare the performances obtained for Case 1 reported in Figure 5a,b (graph a), and the performance for Case 2 reported in Figure 6a,b (graph b). Figure 7 suggests the following: (1) for both the MAE and the MARE, the RF performance curves resulting from the PFI/Shap feature importance lie below (i.e., are better than) the curve obtained with $\kappa^{ALE}$; (2) similarly, for $R^{2}$, the RF performance curve resulting from the PFI/Shap feature importance is higher (i.e., better) than the curve obtained with $\kappa^{ALE}$. Based on these results, it can be concluded that the importance ranking obtained with PFI/Shap offers better performance across all three indices (MAE, MARE, and $R^{2}$). Therefore, it is reasonable to prefer the importance ranking derived from PFI/Shap over the ranking obtained with $\kappa^{ALE}$. Therefore, the most influential features in predicting CVD and RD are *P_atm*, *Tmin*, *CO*, and *rh*.

The analysis identified minimum temperature, atmospheric pressure, and carbon monoxide as the most influential environmental factors predicting cardiovascular and respiratory disease admissions using Random Forest (RF) and feature importance measures (PFI, SHAP, $\kappa^{ALE}$). The RF model demonstrated robust predictive performance, as evidenced by low mean absolute errors for both types of diseases. Our results indicate that these environmental factors play critical roles in influencing health outcomes. Higher minimum temperatures have been linked to increased stress on the cardiovascular system, leading to higher hospital admission rates. Changes in atmospheric pressure can affect blood pressure and respiratory function, while carbon monoxide, a known pollutant, exacerbates respiratory conditions.

These findings align with previous studies. For instance, temperature and atmospheric pressure have already been identified as significant predictors of cardiovascular admissions [1]. Similarly, other research works [2,3] observed significant correlations between temperature fluctuations and mortality rates due to cardiovascular and respiratory diseases. The identification of carbon monoxide as a critical factor was supported by previous studies that highlighted the compounding effects of air pollution and temperature on health [4]. However, some discrepancies were noted. While our study emphasized the role of minimum temperature, other studies [5] focused on maximum temperatures and their effects. These differences underscore the importance of context-specific analyses and the need for localized health intervention strategies. In the context of atmospheric pressure, previous research [5,6] has highlighted its impact on cardiovascular health. These studies often employed linear models, which may not capture the complex, non-linear relationships between atmospheric pressure and health outcomes. Our application of the RF model addresses this limitation by effectively modeling these non-linear interactions, leading to more accurate predictions. When comparing air pollution studies with previous studies [4,7], our analysis with feature importance measures like PFI, SHAP, and $\kappa^{ALE}$ offers a more detailed and flexible analysis of the impacts of pollutants like carbon monoxide. These techniques allowed us to quantify the global and local importance of each feature, providing deeper insights into their specific contributions to health outcomes. Our methodological advancements, particularly the integration of Seasonal Trend Decomposition using LOESS (STL) for data pre-processing, further differentiate our study. STL enhances the robustness and accuracy of our predictions by isolating trend components from seasonal and irregular noise, a step not commonly implemented in previous studies.

The broader implications of our findings suggest significant opportunities for public health policy. Developing early warning systems that integrate these key environmental factors could enhance preparedness and response strategies for cardiovascular and respiratory health risks. Moreover, our study underscores the value of advanced statistical and machine learning techniques in public health research, providing more nuanced insights into the environmental determinants of health. Our results advocate for the incorporation of such predictive models into public health planning to mitigate the adverse health impacts associated with climate change. Future research should expand the range of environmental variables considered and explore these relationships across different geographical locations and populations to validate and extend our findings.

## 5. Conclusions

This study successfully employed permutation feature importance (PFI), Shap feature importance (Shap), and derivative-based importance measure $\kappa^{ALE}$ to identify the most influential climatic features in predicting cardiovascular (CVD) and respiratory (RD) diseases. The findings demonstrate an effective relationship between atmospheric pressure (*P_atm*), minimum temperature (*Tmin*), and carbon monoxide (*CO*) and the incidence of both CVDs and RDs. 

Upon evaluating the performance of several RF configurations built using the feature importance ranking, a notable enhancement in ML model performance was observed. The performance of these configurations significantly improved when incorporating the most influential environmental factors. However, the improvement was only marginal when including the least influential factors. This finding highlights the practical value of utilizing feature importance measures in health and environmental applications to identify the key factors that drive the predictions of ML models.

The application of advanced machine learning techniques, specifically RF and feature importance measures, in the context of environmental health represents a significant theoretical advancement. This study not only reinforces the existing understanding of the relationship between environmental factors and health outcomes, but also introduces a robust methodological framework for future research. By employing feature importance measures, we provide a comprehensive analysis adaptable to various machine learning models and datasets.

The integration of Seasonal Trend Decomposition using LOESS (STL) for data pre-processing, combined with advanced feature importance analysis, underscores the novelty of our approach. This methodology enhances the predictive accuracy of health outcomes by effectively isolating trend components from seasonal and irregular noise, leading to clearer and more reliable model interpretations.

This approach demonstrates how advanced data pre-processing techniques like STL can significantly improve the robustness and accuracy of predictive models. By decomposing complex time-series data into trend, seasonal, and residual components, STL allows for a more nuanced understanding of underlying patterns, which is crucial for accurately modeling environmental health impacts. The use of feature importance measures further strengthens our methodological framework by offering flexibility across various machine learning algorithms. This adaptability is vital in the dynamic field of environmental health research, where data characteristics and modeling needs can vary widely.

Moreover, pinpointing specific environmental factors as major contributors to health risks underscores the potential of machine learning tools to inform and transform public health strategies. The ability to accurately identify and rank the importance of these factors provides actionable insights for policymakers and healthcare providers, facilitating the development of targeted interventions to mitigate adverse health outcomes. This study exemplifies how combining sophisticated machine learning techniques with advanced pre-processing methods can lead to significant advancements in both theoretical understanding and practical application in public health.

Practically, the results of this study have important implications for public health policy and practice. The identification of key environmental predictors allows for the development of more accurate early warning systems that can anticipate spikes in disease admissions, thereby enabling timely public health interventions. Furthermore, these findings can inform the design of targeted strategies to mitigate the health impacts of climate change, particularly in urban areas where pollution and temperature variations are more pronounced.

In summary, this study contributes to both theory and practice by providing a novel methodological approach to understanding and predicting the health impacts of environmental factors. The insights gained from this research have the potential to drive significant advancements in public health policy, particularly in the context of climate change and environmental health. 

## Figures and Tables

**Table 1:** Summary statistics of the environmental factors—average daily minimum temperature (Tmin), average daily atmospheric pressure (P_atm), average daily relative humidity (rh), carbon monoxide (CO), ozone (o3), particulate matter (pm10), and nitrogen dioxide (NO2)—and of cardiovascular (CVD) and respiratory (RD) daily emergency room admissions.

	Tmin [°C]	P_atm [hPa]	rh [%]	CO [mg/m^3^]	o3 [µg/m^3^]	pm10 [µg/m^3^]	NO2 [µg/m^3^]	CVD [Cases/Day]	RD [Cases/Day]
Mean	17.45	1009.56	70.61	0.84	83.15	22.74	53.35	14.94	5.64
std	6.33	8.20	10.96	0.42	21.63	10.89	25.76	5.42	3.23
Min	−0.17	976.60	25,49	0.10	13.00	2.00	5.00	0.00	0.00
25%	12.10	1004.20	63.19	0.50	67.00	15.00	34.00	11.00	3.00
50%	17,16	1009.58	71.15	0.80	82.00	21.00	50.00	14.00	5.00
75%	22.79	1015.29	78.38	1.00	99.00	27.00	70.00	18.00	7.00
Max	32.86	1033.67	99.00	3.00	154.00	117.00	157.00	37.00	25.00

## Data Availability

The data that support the findings of this study are available on request from the corresponding authors.
